# Supplementary material for: Chicken Skin Decontamination of Thermotolerant Campylobacter spp. and Hygiene Indicator Escherichia coli Assessed by Viability Real-Time PCR
Source: Pathogens. 2022 Jun 18;11(6):706. doi: 10.3390/pathogens11060706 (PMC9230925; doi:10.3390/pathogens11060706)
Supplement: Supplementary file 1 [file pathogens-11-00706-s001.zip › pathogens-1721940-supplementary.pdf]

pathogens

MDPI

Supplementary materials

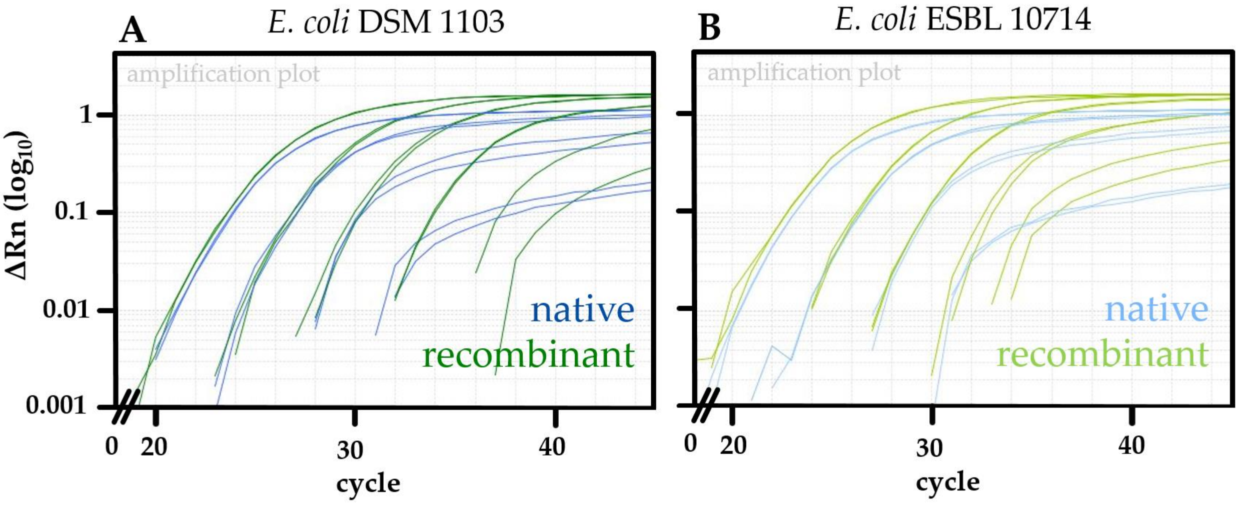

Figure S1. Standard amplification curves obtained with the recombinant versus native Taq polymerases. The different amplification efficiency of the tested enzymes becomes apparent at lower copy numbers. Amplification of five decimal dilutions of 50.000 to 5 genomic copies/reaction are depicted. Green, recombinant Platinum Taq polymerase; blue, native *Thermophilus aquaticus* polymerase. (**A**) *E. coli* DSM1103 DNA standard (**B**) *E. coli* ESBL 10714 DNA standard.

Table S1. List of field isolates tested for sensitivity and specificity of the *uidA*-based qPCR.

Species	BfR strain No.	Serotype	Characteristics	Origin	Product	MUG
Aeromonas jandaei	BfR-EC-16179	O56		chicken	meat	-
Citrobacter koseri	BfR-EC-16493	ONT:[HNT]	nd	cattle	milk	-
Enterobacter cloacae complex	BfR-EC-15955	ONT:[HNT]	nd	cattle	milk	-
Escherichia albertii	BfR-EC-16090	O65:[HNT]	diarrhea	human	clinical	-
Escherichia albertii	BfR-EC-16091	ONT/O127	diarrhea	human	clinical	-
Escherichia coli	*E. coli* 10714 (BfR-CA-15244)	nd	TEM-52	chicken	meat	+
Escherichia coli	BfR-EC-11061	ONT:H7	commensal	chicken	faeces	+
Escherichia coli	BfR-EC-11062	ONT:H4	commensal	chicken	faeces	+
Escherichia coli	BfR-EC-11063	O78:NM	commensal	chicken	faeces	+
Escherichia coli	BfR-EC-11734	O157:NM	commensal	chicken	meat	+
Escherichia coli	BfR-EC-11735	O157:NM	commensal	chicken	meat	+
Escherichia coli	BfR-EC-11755	O68:NM	commensal	chicken	meat	+
Escherichia coli	BfR-EC-11756	O182:NM	commensal	chicken	meat	+
Escherichia coli	BfR-EC-11757	O120:H4	commensal	chicken	meat	+
Escherichia coli	BfR-EC-11758	O120:H4	commensal	chicken	meat	+
Escherichia coli	BfR-EC-11759	O9:NM	commensal	chicken	meat	+
Escherichia coli	BfR-EC-11760	O78:NM	commensal	chicken	meat	+
Escherichia coli	BfR-EC-11842	O78:NM	commensal	goose	inner organs	+
Escherichia coli	BfR-EC-11893	O22:H7	commensal	chicken	meat	+
Escherichia coli	BfR-EC-11894	O22:H2	commensal	chicken	meat	+

Escherichia coli	BfR-EC-12047	O45:[H2]	STEC, stx1+	unknown	unknown	(+)
Escherichia coli	BfR-EC-12481	O153:H12	commensal	chicken	faeces	+
Escherichia coli	BfR-EC-12482	O153:H12	commensal	chicken	faeces	+
Escherichia coli	BfR-EC-12499	O103:[H11]	STEC, stx1+	cattle	faeces	+
Escherichia coli	BfR-EC-13091	O88:H8	commensal	chicken	meat	+
Escherichia coli	BfR-EC-13092	Or:H19	commensal	chicken	meat	+
Escherichia coli	BfR-EC-13093	Or:H4	commensal	chicken	meat	(+)
Escherichia coli	BfR-EC-13094	Or:H4	commensal	chicken	meat	+
Escherichia coli	BfR-EC-13095	O1:H7	commensal	chicken	meat	+
Escherichia coli	BfR-EC-13096	O2:H9	commensal	chicken	meat	+
Escherichia coli	BfR-EC-13097	O78/149:H4	commensal	chicken	meat	(+)
Escherichia coli	BfR-EC-13098	Or:H4	commensal	chicken	meat	+
Escherichia coli	BfR-EC-13099	O16:H11	commensal	chicken	unknown	-
Escherichia coli	BfR-EC-13216	O136:H40	EPEC	chicken	meat	+
Escherichia coli	BfR-EC-13217	O9:H10	EPEC	chicken	meat	+
Escherichia coli	BfR-EC-13218	O103:H2	EPEC	chicken	meat	+
Escherichia coli	BfR-EC-13573	O89:[H8]	commensal	turkey	meat	+
Escherichia coli	BfR-EC-13574	O103:[H16]	commensal	turkey	meat	+
Escherichia coli	BfR-EC-13575	O15:[H27]	STEC, stx1+	turkey	meat	+
Escherichia coli	BfR-EC-13581	O9:[H51]	commensal	turkey	meat	-
Escherichia coli	BfR-EC-13616	O8:[H25]	commensal	chicken	unknown	-
Escherichia coli	BfR-EC-13671	O82:H40	EPEC	chicken	meat	+
Escherichia coli	BfR-EC-13702	O82:H40	EPEC	chicken	meat	+
Escherichia coli	BfR-EC-13703	O82:H40	EPEC	chicken	meat	+
Escherichia coli	BfR-EC-13712	O157:[H7]	EPEC	cattle	meat	+
Escherichia coli	BfR-EC-13713	O157:[H7]	STEC, stx2+	cattle	meat	+
Escherichia coli	BfR-EC-13714	O157:[H7]	STEC, stx2+	cattle	meat	+
Escherichia coli	BfR-EC-13715	O157:[H7]	STEC, stx2+	cattle	meat	+
Escherichia coli	BfR-EC-13716	O157:[H7]	STEC, stx2+	cattle	meat	+
Escherichia coli	BfR-EC-13717	O157:[H7]	STEC, stx2+	cattle	meat	+
Escherichia coli	BfR-EC-13718	O157:[H7]	EPEC	cattle	meat	+
Escherichia coli	BfR-EC-13813	O113:H6	EPEC	turkey	meat	+
Escherichia coli	BfR-EC-13814	O113:H6	EPEC	turkey	meat	+
Escherichia coli	BfR-EC-13938	O80:NM	EPEC	chicken	meat	+
Escherichia coli	BfR-EC-13946	O186:H40	EPEC	chicken	meat	+
Escherichia coli	BfR-EC-13947	O186:H40	EPEC	chicken	meat	+
Escherichia coli	BfR-EC-13948	O186:H40	EPEC	chicken	meat	+
Escherichia coli	BfR-EC-14612	O157:[H7]	STEC, stx1+stx2+	wild animals	meat	-
Escherichia coli	BfR-EC-14642	O45:[H2]	STEC, stx1+	unknown	unknown	-
Escherichia coli	BfR-EC-14794	O136:H40	EPEC	chicken	inner organs	+
Escherichia coli	BfR-EC-14795	O136:H40	EPEC	chicken	meat	+
Escherichia coli	BfR-EC-14815	O136:H40	EPEC	chicken	meat	+
Escherichia coli	BfR-EC-14816	O91:H40	EPEC	chicken	meat	+
Escherichia coli	BfR-EC-14817	Or:H40	EPEC	chicken	meat	+
Escherichia coli	BfR-EC-14883	O16:NM	commensal	chicken	meat	+
Escherichia coli	BfR-EC-14899	O157:[H7]	STEC, stx1+stx2+	cattle	faeces	-
Escherichia coli	BfR-EC-14910	ONT:[H26]	EPEC	chicken	meat	-
Escherichia coli	BfR-EC-14920	O157:[H7]	STEC, stx2+	unknown	red minced meat	-

Escherichia coli	BfR-EC-14955	O157:[H7]	STEC, stx2+	cattle	milk	-
Escherichia coli	BfR-EC-15046	O80:r	EPEC	chicken	meat	-
Escherichia coli	BfR-EC-15050	O145:[H40]	EPEC	chicken	meat	+
Escherichia coli	BfR-EC-15051	O68:H28	EPEC	chicken	meat	-
Escherichia coli	BfR-EC-15052	O26:H31	EPEC	chicken	meat	+
Escherichia coli	BfR-EC-15054	O82:H40	EPEC	chicken	meat	+
Escherichia coli	BfR-EC-15058	Or:H10	EPEC	chicken	meat	+
Escherichia coli	BfR-EC-15059	ONT:NT	EPEC	chicken	meat	-
Escherichia coli	BfR-EC-15061	O111:[H25]	STEC, stx2+	cattle	milk	-
Escherichia coli	BfR-EC-15083	O157:[H7]	STEC, stx2+	cattle	faeces	-
Escherichia coli	BfR-EC-15108	O111:[H29]	STEC, stx2+	cattle	meat	+
Escherichia coli	BfR-EC-15112	O51:[H40]	EPEC	turkey	meat	+
Escherichia coli	BfR-EC-15113	ONT:H10	EPEC	chicken	inner organs	(+)
Escherichia coli	BfR-EC-15118	O111:[H8]	EPEC	plant	plant	+
Escherichia coli	BfR-EC-15120	O40:H45	EPEC	chicken	meat	+
Escherichia coli	BfR-EC-15123	O2:H40	EPEC	chicken	meat	+
Escherichia coli	BfR-EC-15127	O45:NM	EPEC	chicken	meat	+
Escherichia coli	BfR-EC-15128	O2:H40	EPEC	chicken	meat	+
Escherichia coli	BfR-EC-15172	O145:H31	commensal	cattle	faeces	+
Escherichia coli	BfR-EC-15190	O123:H40	EPEC	chicken	meat	+
Escherichia coli	BfR-EC-15238	O157:[H7]	STEC, stx1+stx2+	cattle	faeces	-
Escherichia coli	BfR-EC-15265	ONT:H40	EPEC	chicken	meat	+
Escherichia coli	BfR-EC-15268	O123/186:H40	EPEC	chicken	meat	+
Escherichia coli	BfR-EC-15272	O61:NM	EPEC	chicken	meat	+
Escherichia coli	BfR-EC-15273	O145:H40	EPEC	chicken	meat	+
Escherichia coli	BfR-EC-15374	ONT:H40	EPEC	chicken	meat	+
Escherichia coli	BfR-EC-15375	O6:H49	EPEC	chicken	meat	+
Escherichia coli	BfR-EC-15378	O145:[H40]	EPEC	chicken	meat	+
Escherichia coli	BfR-EC-15384	O121:[H19]	STEC, stx1+	sheep	milk	+
Escherichia coli	BfR-EC-15386	ONT:H10	EPEC	chicken	meat	+
Escherichia coli	BfR-EC-15387	O80:H26	EPEC	chicken	meat	-
Escherichia coli	BfR-EC-15389	O108/130:H9	EPEC	chicken	meat	+
Escherichia coli	BfR-EC-15393	O71:H40	EPEC	chicken	meat	-
Escherichia coli	BfR-EC-15394	O123/186:H40	EPEC	chicken	meat	+
Escherichia coli	BfR-EC-15417	O19:H28	commensal	goose	inner organs	+
Escherichia coli	BfR-EC-15421	O154:H16	commensal	goose	unknown	+
Escherichia coli	BfR-EC-15502	O123/ 186:H40	EPEC	chicken	meat	+
Escherichia coli	BfR-EC-15564	O145:[H28]	EPEC	domestic pig	faeces	+
Escherichia coli	BfR-EC-15566	O145:[H28]	EPEC	domestic pig	faeces	+
Escherichia coli	BfR-EC-15652	O157:[H7]	STEC, stx1+stx2+	cattle	faeces	-
Escherichia coli	BfR-EC-15812	O157:[H7]	STEC, stx2+	cattle	cheese	-
Escherichia coli	BfR-EC-15938	O157:[H7]	STEC, stx2+	cattle	milk	-
Escherichia coli	BfR-EC-15942	O103:[H2]	STEC, stx1+	cattle	meat	+
Escherichia coli	BfR-EC-15972	O157:[H7]	STEC, stx1+stx2+	cattle	faeces	-
Escherichia coli	BfR-EC-16053	O103:[H2]	STEC, stx1+	cattle	faeces	(+)
Escherichia coli	BfR-EC-16463	O2:H5	commensal	chicken	meat	+
Escherichia coli	BfR-EC-16464	O33:H10	commensal	chicken	meat	+

Escherichia coli	BfR-EC-16465	ONT:NM	commensal	chicken	meat	+
Escherichia coli	BfR-EC-16466	O33:H10	commensal	chicken	meat	+
Escherichia coli	BfR-EC-16467	O33:H10	commensal	chicken	meat	+
Escherichia coli	BfR-EC-16468	O33:H10	commensal	chicken	meat	+
Escherichia coli	BfR-EC-16469	O33:H10	commensal	chicken	meat	+
Escherichia coli	BfR-EC-16470	O33:H10	commensal	chicken	meat	+
Escherichia coli	BfR-EC-16627	O26:[H11]	STEC, stx1+	cattle	cheese	(+)
Escherichia coli	BfR-EC-16636	O121:[H19]	EPEC	wild animals	meat	+
Escherichia coli	BfR-EC-16666	O103:[H2]	STEC, stx1+	cattle	meat	+
Escherichia coli	BfR-EC-16692	O45:[H2]	STEC, stx2+	wild animals	faeces	+
Escherichia coli	BfR-EC-16734	O121:[H10]	STEC, stx2+	domestic pig	meat	+
Escherichia coli	BfR-EC-16972	O103:[H2]	STEC, stx1+	cattle	meat	(+)
Escherichia coli	BfR-EC-16975	O87:[H16]	STEC, stx2+	chicken	egg	+
Escherichia coli	BfR-EC-17175	O145:[H28]	STEC, stx2+	plant	plant	+
Escherichia coli	BfR-EC-17218	O145:[H28]	STEC, stx2+	cattle	cheese	+
Escherichia coli	BfR-EC-17223	O121:[H14]	STEC, stx1+	cattle	meat	+
Escherichia coli	BfR-EC-17240	O26:[H11]	STEC, stx1+	wild animals	faeces	+
Escherichia coli	BfR-EC-17596	O54:[H21]	STEC, stx2e+	turkey	raw sausage	+
Escherichia coli	BfR-EC-17618	O26:[H11]	STEC, stx1+	cattle	milk	+
Escherichia coli	BfR-EC-17761	O121:[H19]	EPEC	plant	plant	+
Escherichia coli	BfR-EC-17783	O98:[H21]	STEC, stx1+	cattle	milk	(+)
Escherichia coli	BfR-EC-17804	O26:[H11]	EPEC	cattle	cheese	+
Escherichia coli	BfR-EC-17836	O26:[H11]	STEC, stx2+	cattle	cheese	+
Escherichia coli	BfR-EC-17944	O113:[H21]	STEC, stx2+	cattle	meat	(+)
Escherichia coli	BfR-EC-17949	O109:[H16]	STEC, stx1+	cattle	milk	(+)
Escherichia coli	BfR-EC-17971	O103:[H2]	STEC, stx1+	cattle	faeces	+
Escherichia coli	BfR-EC-17972	O111:[H29]	STEC, stx1+	cattle	faeces	+
Escherichia coli	BfR-EC-17986	O8:[H9]	STEC, stx2+	domestic pig	meat	(+)
Escherichia coli	BfR-EC-18006	O184:[H2]	STEC, stx1+	cattle	faeces	(+)
Escherichia coli	BfR-EC-18009	O26:[H11]	STEC, stx1+	cattle	faeces	+
Escherichia coli	BfR-EC-18019	O157:[H7]	STEC, stx1+stx2+	plant	plant	-
Escherichia coli	BfR-EC-18033	nd	EPEC	cattle	appendix content	+
Hafnia alvei	BfR-EC-15818	ONT:HNM	nd	cattle	cheese (from raw milk)	-
Hafnia alvei	BfR-EC-15819	Or:HNM	nd	cattle	cheese (from raw milk)	-
Hafnia alvei	BfR-EC-15932	O91:HNM	nd	wild animals	meat	-

nd, not determined

Table S2: Absence of false-positive *uidA* signals in negative controls using different Taq Polymerases and the amplification control IPC-ntb2.

Sample name	Experiment ID	Taq Polymerase	U or x-fold /reaction	Ct of *uidA*	Ct of IPC-ntb2
1000 copies IPC-ntb2	1	Platinum Taq	2	undetermined	28,94
1000 copies IPC-ntb2	1	Platinum Taq	2	undetermined	29,24
25 copies IPC-ntb2	2	Platinum Taq	2	undetermined	34,07
25 copies IPC-ntb2	2	Platinum Taq	2	undetermined	34,60
25 copies IPC-ntb2	3	Platinum Taq	2	undetermined	34,29
25 copies IPC-ntb2	3	Platinum Taq	2	undetermined	34,11
25 copies IPC-ntb2	4	Platinum Taq	2	undetermined	33,54
25 copies IPC-ntb2	4	Platinum Taq	2	undetermined	33,56
25 copies IPC-ntb2	5	Platinum Taq	2	undetermined	34,88
25 copies IPC-ntb2	5	Platinum Taq	2	undetermined	34,63
25 copies IPC-ntb2	6	Platinum Taq	2	undetermined	34,23
25 copies IPC-ntb2	6	Platinum Taq	2	undetermined	34,11
25 copies IPC-ntb2	7	Platinum Taq	2	undetermined	35,23
25 copies IPC-ntb2	7	Platinum Taq	2	undetermined	34,59
25 copies IPC-ntb2	8	Platinum Taq	2	undetermined	34,94
25 copies IPC-ntb2	8	Platinum Taq	2	undetermined	34,22
25 copies IPC-ntb2	9	Platinum Taq	2	undetermined	32,27
25 copies IPC-ntb2	9	Platinum Taq	2	undetermined	33,22
25 copies IPC-ntb2	10	Platinum Taq	2	undetermined	33,75
25 copies IPC-ntb2	10	Platinum Taq	2	undetermined	34,41
25 copies IPC-ntb2	11	Platinum Taq	2	undetermined	33,05
25 copies IPC-ntb2	11	Platinum Taq	2	undetermined	34,04
25 copies IPC-ntb2	12	TaqMan Gene Expression Master Mix	2x	undetermined	36,79
25 copies IPC-ntb2	12	Quanti Tect Multiplex PCR Master Mix	2x	undetermined	34,63
25 copies IPC-ntb2	13	Quanti Tect Multiplex PCR Master Mix	2x	undetermined	34,67
25 copies IPC-ntb2	13	TaqMan Gene Expression Master Mix	2x	undetermined	38,65
25 copies IPC-ntb2	14	TaqMan Gene Expression Master Mix	2x	undetermined	37,19
25 copies IPC-ntb2	15	TaqMan Gene Expression Master Mix	2x	undetermined	34,92
25 copies IPC-ntb2	16	Platinum Taq	2	Undetermined	34,49
25 copies IPC-ntb2	16	Platinum Taq	4	Undetermined	35,19
25 copies IPC-ntb2	16	Platinum Taq	10	Undetermined	34,93
25 copies IPC-ntb2	16	Platinum Taq	20	Undetermined	35,21

All qPCR runs contained positive control samples, which amplified the target *uidA*-P-JOE.
